# Jean-Jacques Muyembe Tamfum: a life’s work on Ebola 

**DOI:** 10.2471/BLT.18.031218

**Published:** 2018-12-01

## Abstract

Jean-Jacques Muyembe Tamfum was part of the research team that investigated the first known outbreak of Ebola virus disease in 1976. He talks to Fiona Fleck about those experiences and how he and his colleagues are using the knowledge they have built up in recent Ebola outbreaks.

Q: How did you become interested in epidemiology?

A: After graduating in medicine, I decided to do a PhD in virology at the University of Leuven in Belgium where I started to do some research in the treatment of viral infections, working with mice, but when I went back to Congo, at that time called Zaïre, I was unable to pursue my work, because there were no labs there and no laboratory animals. That year, 1974, there was a cholera outbreak in the Port of Matadi, and I was dispatched to investigate. The following year I was sent to investigate an outbreak of bacterial meningitis that was killing a lot of soldiers in the military camp of Kitona in the Kongo central province. I went to the location, and isolated the bacteria. The government launched a vaccination campaign, and that was the end of the epidemic.

Q: So was that how you started to work on outbreak investigations?

A: Yes, those two experiences made me realise that I couldn’t just study microbes in the laboratory, but needed to get into the field. Then, in 1976, there was an outbreak of a mysterious disease at a Catholic mission run by Belgian nuns in Yambuku, in the north of the country. The health minister sent me and Dr K Omombo to take a look. Yambuku was a remote village in the forest and when I arrived, the place was deserted. It was as if nobody lived there. It was the same at the hospital. Most of the nurses were dead, and all the patients had fled except for one patient, a child. The mother said the child had malaria, but then the child died in the night. The next day villagers showed up at the hospital. They’d heard that we had come from Kinshasa with medicine. Many of them had fever and diarrhoea. I thought they perhaps had typhoid fever, and I collected blood samples. But I noticed that when I removed the needle from people’s arms, the puncture wounds bled a lot. My fingers and hands were covered in blood. So I used water and soap to wash it off.

“If I had not washed my hands, I would have died.”

Q: You didn’t wear gloves?

A: Back then, we used our bare hands, we had no protective clothing. Later, I took some liver samples from two corpses, using a steel rod and so of course there was even more blood, and again I washed with soap and water. The liver biopsies were inconclusive, but then I examined a Belgian nun who had developed a fever, and I said to her, “since we don't know how to diagnose this disease, I'm going to take you to Kinshasa.” She said: “I can't leave because they're going to think that I'm escaping because of the disease.” Finally, we persuaded her and we left. When we got to Kinshasa, we took a blood sample from her and sent it to the Institute of Tropical Medicine in Antwerp (Belgium) where Peter Piot worked. It was from the blood of this nun that Piot first isolated the Ebola virus.

Q: It’s amazing that you survived.

A: Yes, it is extraordinary. If I had not washed my hands, I would have died. After Yambuku there were no more outbreaks for a long time. Then, in 1995, I got a call from the director of Kikwit General Hospital saying that there had been an outbreak of bloody diarrhoea that had already caused several deaths. I also got a message from the Diocese of Kikwit asking me to come and help. I suspected that it was shigellosis or something like that and that I could resolve the problem quickly, so I left with only two pairs of trousers. When I got there, however, and had a look around, I realised it was not shigellosis. It was Ebola. And Kikwit was different to Yambuku; this was a town, not a village, so the risk of the disease spreading was far greater. People said to me, how can it be Ebola? We are at least 1000 kilometres away from Yambuku. But I was quite sure. So, I collected samples and sent them to the Centers for Disease Prevention and Control through the Institute of Tropical Medicine in Antwerp. Two days later, they confirmed that it was Ebola.

Q: What happened then?

A: There was complete panic, but I started setting up teams to care for the patients at the hospital. I met with a group of doctors and asked who was ready to go into quarantine with the patients. Nobody looked at me, but after a few minutes one of them got up and said “I am.” He was a young doctor. I said “Are you sure? You will be put in isolation and won’t be able to leave for at least 48 hours.” He agreed. So did the nurses. That was the beginning of the emergency response. Then WHO was notified and sent experts led by Dr David Heymann to join us. I led the response to the epidemic, which eventually killed at least 254 people. Since then, of course, more Ebola outbreaks have occurred in my country, in 2007, 2008, 2009, 2012, 2014, 2017 and now two in 2018. I have been present at all of them. I’ve spent all my life and my entire career fighting Ebola.

Q: How do you use the knowledge you’ve acquired over all these years to fight against the Ebola epidemic today?

A: I and my colleagues have given presentations at more than 50 conferences. We’ve also published a number of papers. However, my most significant contribution to the fight against Ebola virus disease may be my work on the use of antibodies as the basis for therapeutic medicines, a line of inquiry that is based on observations made during the Kikwit epidemic. My team collected blood from Ebola survivors in Kikwit and gave it to eight patients infected with the virus. Seven of those patients recovered, suggesting the antibodies in convalescent blood acted as a protection. This idea was not accepted by most scientists at the time, but I remained convinced that antibodies could work, so in 2004 I took one of the survivors of the Kikwit epidemic and sent him to the United States of America, where colleagues, including Dr Nancy Sullivan, collected some blood cells from him and cultured them. They managed to produce monoclonal antibodies and gave them to monkeys infected with Ebola. The monkeys all recovered.

Q: There is an ongoing discussion about five experimental therapeutics.

A: Yes, and these studies with antibodies and monkeys led to one of them – mAB 114. So that is certainly an important contribution to finding an effective and safe treatment for Ebola. However, now in the Democratic Republic of the Congo we are focusing on research into the reservoirs of the virus, because we still don’t know where Ebola comes from. For a long time we thought that bats were the reservoir, but we’ve examined thousands of samples and haven’t found it. So we must continue to search.

“The biggest challenge is the security situation.”

Q: You have also helped set up research facilities. Can you tell us about how you came to participate in the construction of your research institute?

A: That is a long story. It starts back in 1975 when the Minister of Health asked me to develop the design for an institute like the Pasteur Institute in Paris. At first I was only involved in the design process. Then in 1998, I was appointed director-general of our National Institute for Biomedical Research. WHO set up its polio research laboratory here in 1988 and then the CDC added its influenza lab. I also got a mobile lab which has been paid for by United States Agency for International Development. Last year I signed a contract with the Japan International Cooperation Agency (JICA) to build a US$ 23 million state-of-the-art lab complex, which will include a centre for clinical tests, a conference centre, three biosafety level 3 labs (suitable for work with pathogens which can cause serious/lethal diseases via the inhalation route), and three biosafety level 2 labs (suitable for work with pathogens representing moderate potential hazard). It will be fantastic.

Q: Do you have a lab where you can test for Ebola?

A: We do, but it is not adequate. To date none of my lab technicians or doctors has been infected, which is very important. With the JICA investment we’ll have a high security lab (biosafety level 3) where we can work safely with Ebola samples.

Q: What are the challenges for improving the detection of Ebola in rural areas?

A: Well to begin with our country is huge, about 2.4 million square kilometres, much of it inaccessible by road. So even without the kind of conflict and instability that we have experienced, surveillance is a challenge. The country is divided into 516 health zones and we have a surveillance system in each zone. Community volunteers report health events in their villages, and these reports are collected at the central level. When a health event is considered potentially significant, the province dispatches epidemiologists who can investigate and collect samples. Unfortunately, it can take a week for the sample to arrive at our institute. That’s why we are planning to build labs in each province.

Q: What are the main challenges with the current Ebola outbreak?

A: We are facing many challenges to end this outbreak. The biggest challenge is the security situation, which is characterized by the presence of several active armed groups, the lack of community engagement in some places, and the geographic spread of the transmission in multiple foci.

Q: How is your country managing the outbreak?

A: We have put in place strategies that have been successful in previous outbreaks: early detection of cases, contact tracing, community engagement, safe burial, clinical management of symptoms and psychosocial care. We have also added two new tools since the May 2018 outbreak: vaccination and the use of experimental therapeutics. For the first time we are offering these therapeutics to patients infected with the Ebola virus in the Democratic Republic of the Congo under a compassionate-use protocol and planning to evaluate their efficacy in a randomized controlled trial.

